# Navigation-Guided C-arm-Free Minimally Invasive Transforaminal Lumbar Interbody Fusion: A Comparative Study of Cage Orientation and Screw Insertion Accuracy Against the Conventional C-arm-Assisted Technique

**DOI:** 10.7759/cureus.66070

**Published:** 2024-08-03

**Authors:** Koji Uotani, Masato Tanaka, Chetan Kumawat, Sharvari Gunjotikar, Yoshiaki Oda, Kensuke Shinohara, Tadashi Komatsubara, Shinya Arataki, Toshifumi Ozaki

**Affiliations:** 1 Department of Orthopaedic Surgery, Okayama University Hospital, Okayama, JPN; 2 Department of Orthopaedic Surgery, Okayama Rosai Hospital, Okayama, JPN; 3 Department of Orthopaedic Surgery, Okayama University Graduate School of Medicine, Dentistry and Pharmaceutical Sciences, Okayama, JPN

**Keywords:** navigation, o-arm, c-arm free, mis-tlif, spine surgery

## Abstract

Background: Minimally invasive transforaminal lumbar interbody fusion (MIS-TLIF) is a widely utilized technique in spine surgery. This study compares the efficacy and safety of MIS-TLIF performed with traditional C-arm fluoroscopy and C-arm-free O-arm navigation. To the best of our knowledge, our study is the first to compare cage positioning between C-arm-free and C-arm techniques for MIS- TLIF.

Methods: A retrospective, comparative analysis was conducted on 43 patients undergoing MIS-TLIF. The group was divided based on the utilization of C-arm fluoroscopy or C-arm-free O-arm navigation. Key parameters analyzed included cage orientation, screw insertion accuracy, operative efficiency, and postoperative recovery. Radiographic measurements were used to assess surgical precision and perioperative complications were documented.

Results: The study encompassed 43 patients, with no significant differences in demographic characteristics between the two groups. Surgical time and blood loss were comparable between C-arm-free and C-arm groups. O-arm navigation significantly reduced pedicle screw misplacement (p=0.024). Cage positioning differed between groups (p=0.0063): O-arm cages were mostly mid-center, while C-arm cages were more anterior-center. Such differences in the cage location did not cause any impact on clinical outcome. No significant differences were observed in postoperative complications (screw loosenings, dural tears, surgical site infections) between groups. The Oswestry Disability Index scores at the final follow-up showed no significant difference between the O-arm and C-arm groups, indicating similar levels of postoperative disability.

Conclusion: Despite the clinically insignificant difference in cage placement between C-arm-free and C-arm dependent, C-arm-free MIS-TLIF significantly improves screw placement accuracy and reduces radiation exposure to operating stuff. This suggests its potential as a valuable tool for safer and more precise spinal fusion surgery.

## Introduction

From degenerative disc disease to spondylolisthesis, spanning a broad spectrum, these conditions can significantly impair function and quality of life, underscoring the immense challenge we face in managing these debilitating conditions [1]. Previously, transforaminal lumbar interbody fusion (TLIF) has been a common surgical procedure for addressing these conditions, with the goal of alleviating pain, improving spinal stability, and enhancing function. Now, minimally invasive transforaminal lumbar interbody fusion (MIS-TLIF) has emerged as a pivotal technique in spine surgery, offering significant advantages over traditional open approaches [2-4]. This method reduces tissue trauma, blood loss, and postoperative pain, facilitating quicker recovery times for patients with degenerative lumbar disease [5,6]. However, the precision of pedicle screw placement and cage insertion remains a critical concern due to the limited visual field in minimally invasive procedures [7,8]. The advent of imaging guidance systems, particularly the use of intraoperative C-arm, has been a significant advancement in this field. It provides real-time imaging, enabling accurate screw placement and cage alignment [9]. Nonetheless, the use of fluoroscopy raises concerns about the exposure of both patients and surgical teams to ionizing radiation [5]. Prolonged radiation exposure is associated with potential health risks, necessitating the exploration of alternative imaging modalities.

O-arm navigation systems have recently been introduced as a solution to mitigate these concerns. These systems provide three-dimensional intraoperative imaging with reduced radiation exposure, potentially enhancing the accuracy of hardware placement without the associated risks of traditional fluoroscopy [9]. The application of O-arm navigation in MIS-TLIF procedures in spinal surgery potentially combines the benefits of minimally invasive techniques with the precision of instrumentation [10]. The adoption of new technologies like O-arm navigation in MIS-TLIF warrants thorough investigation to establish their efficacy and safety.

Despite the increasing use of navigated and C-arm-guided MIS TLIF, there is a dearth of literature comparing the outcomes of these two methods. Therefore, this study aims to compare the outcomes of MIS-TLIF performed with traditional C-arm fluoroscopy to those utilizing C-arm-free O-arm navigation, focusing on surgical outcomes such as cage orientation and screw insertion accuracy, operative efficiency, and postoperative recovery.

## Materials and methods

Study design

We conducted a retrospective, comparative study of patients undergoing MIS-TLIF. The participants were divided into two groups based on whether the procedure was performed with C-arm-free O-arm navigation or with the use of C-arm fluoroscopic guidance. This investigation adhered strictly to the ethical standards set forth in the Declaration of Helsinki for medical research involving human subjects and received approval from the institutional review board of our hospital.

Patients selection

The inclusion criterion was any patients who underwent one or two level TLIF due to lumbar degenerative diseases. The study encompassed 43 consecutive patients. These procedures were conducted at a single medical institution over the period from April 2017 to April 2019. The exclusion criteria were inaccessibility of data and less than one year of follow-up. Each patient was followed up for a period exceeding one-year post-surgery. Functional outcome was evaluated using the Oswestry Disability Index (ODI) in follow-up periods. Inclusion criteria were (1) lumbar spondylolisthesis grade 1 or 2; (2) Persistent mechanical lower back pain with or without lower limb radiation pain, confirmed by radiologic findings; and (3) Failure of more than three months of conservative treatment, or recurrent attacks of symptom. Exclusion criteria were (1) lumbar spondylolisthesis grade 3 or more; and (2) the presence of infectious diseases, metabolic bone diseases, spinal trauma, deformities, or tumors.

Surgical procedures of C-arm-free O-arm navigation-guided MIS-TLIF

**Figure 1:**
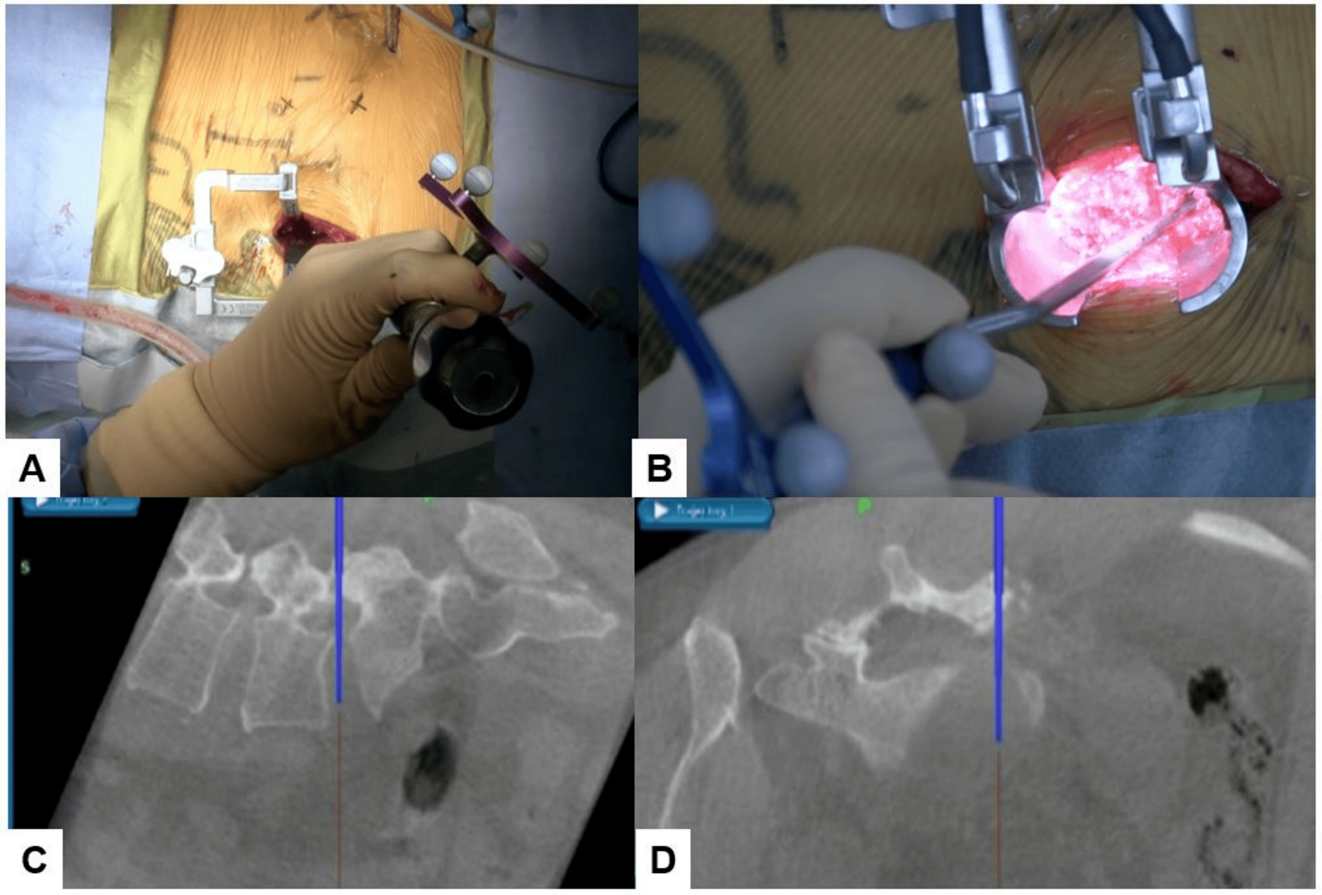
Surgical technique of C-arm-free TLIF Place the reference frame percutaneously into the ilium near the posterior superior iliac spine and capture images with the O-arm. Use the navigation to determine the pedicle screw entry point and create a mini-open incision at the midpoint between the facet joint and the pedicle screw entry point on the side intended for cage insertion. On the cage insertion side, perform tapping up to the screw hole (A); on the contralateral side, insert the pedicle screw. Under navigation, resect the facet joint and intervertebral disc (B), then insert and rotate a banana-shaped cage without the use of a C-arm. After cage placement, use a navigated pointer to approximate the position verification (C). Confirm final positioning with X-ray imaging (D). TLIF: transforaminal lumbar interbody fusion

Patients were placed in a prone position under general anesthesia. A reference frame was attached to the percutaneous pin, which was fixed into the ilium near the posterior superior iliac spine. Following this, an O-arm scan was performed to obtain reconstructed 3D images. Using the navigation system, percutaneous pedicle screws (PPS) were accurately inserted. Skin incisions made for both cranial and caudal PPS were then connected on the symptomatic side. Facet joints were resected using an osteotome, and this local bone graft was used for bone grafting. The disc space was prepared with navigated instruments to ensure appropriate direction and depth. A banana cage was inserted with a bone graft. The end position of the cage was confirmed using a navigated pointer. Finally, rods were anchored to the screws followed by a compression procedure to ensure stability and alignment (Figure 1).

Surgical procedures for MIS-TLIF with C-arm

The same steps were followed in this procedure under the guidance of a C-arm instead of an O-arm. The banana cage was inserted under C-arm guidance.

Radiographic measurements

**Figure 2:**
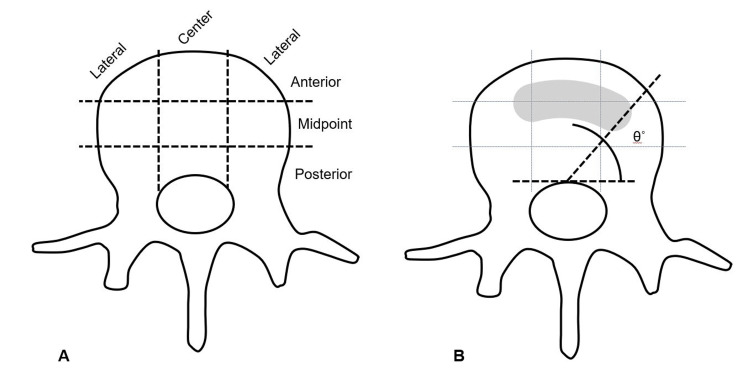
Positioning and orientation of spinal cages (A) An axial view illustration of the vertebral body, segmented into three equal sections in the sagittal and coronal planes. (B) The orientation angle (θ) of the cage is defined by the line extending from the center of the cage to the center of the spinal canal, in relation to a horizontal baseline.

The positioning of the cage was assessed using postoperative CT images. In the axial view, the vertebral body was divided into nine segments by trisecting lines in both the sagittal and coronal planes. Additionally, the rotation of the cage was defined by the angle created between a horizontal baseline and a line extending from the center of the cage to the center of the spinal canal (Figure 2). Screw malposition was defined as Gertzbein-Robbins scale grade C or more (pedicle breach > 2mm) [11].

The presence of screw loosening and non-union were diligently evaluated by two experienced spine surgeons using postoperative functional X-ray and CT images obtained at the final follow-up.

Complications

In assessing complications, the incidence of dural tears, neurological deterioration, epidural hematoma, and surgical site infections were specifically investigated within each group.

Statistical analysis

Ordinal scale data in this study were analyzed using the Mann-Whitney U test, while continuous variables were evaluated using the independent samples t-test, a parametric method ideal for comparing the means of two independent groups. Nominal scale data were compared using Fisher's exact test to determine the presence of nonrandom associations between two categorical variables. All statistical calculations were meticulously performed using GraphPad Prism version 6.0 (GraphPad Software, La Jolla, CA, USA). Statistical significance was set at p<0.05, with this threshold guiding the identification of meaningful differences and associations within the study's findings.

## Results

Patient demographics and characteristics

The study encompassed 43 patients, with mean ages of 73.8 and 65.7 years in the C-arm-free and C-arm groups, respectively. Detailed demographic characteristics, including age and gender distributions, are presented in Table 1. No significant differences in age and gender were observed between the groups.

**Table 1:** Patient demographics The statistical analysis was done by Fisher's exact test and a significant difference was set at p<0.05.

Patient demographics	C-arm free	C-arm	p-value
Number of patients (N)	30	13	-
Age (years)	73.8	65.7	0.144
Sex (M/F)	18/12	7/6	0.747
Total number of Intervertebral disc levels	34	14	-
L2/3	4	1	-
L3/4	3	3	-
L4/5	14	4	-
L5/S	13	6	-
Total number of pedicle screws	136	70	-

Surgical outcomes

**Figure 3:**
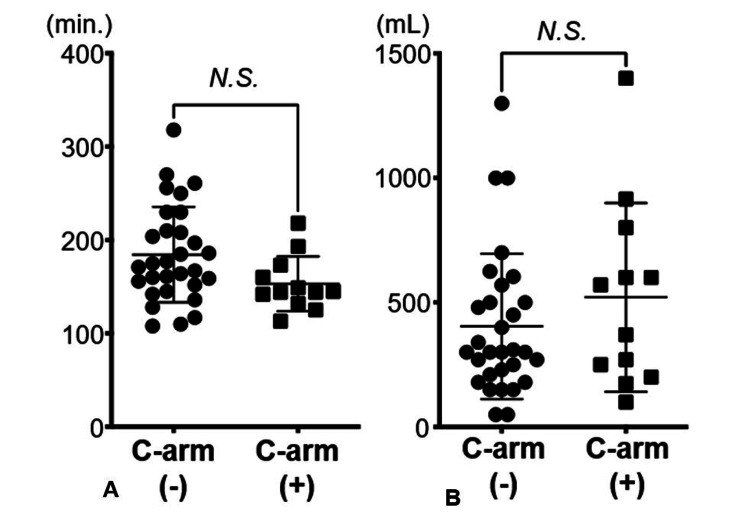
The duration of surgery and estimated blood loss per level There was no significant difference in the duration of surgery (A) and estimated blood loss (B) between the group undergoing surgery without a C-arm and the group undergoing surgery with a C-arm. The statistical analysis was done by the Mann-Whitney U test and a significant difference was set at p<0.05.

The surgical time for the C-arm group was 153.2 minutes, while for the C-arm-free group, it was 184.4 minutes, with no significant difference (p-value=0.054) between them. Similarly, blood loss was comparable between the groups, with the C-arm group experiencing 520.8 mL and the C-arm-free group 416.2 mL (p-value=0.288). These results are depicted in Figure 3.

Comparison of cage positioning

**Figure 4:**
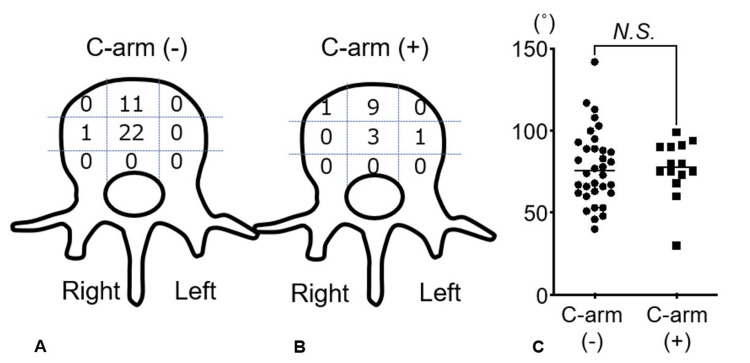
Cage position and rotation analysis (A) Cage positioning in the C-arm-free group, showing a tendency for cages to be placed at the mid-center of the endplate. (B) Cage positioning in the C-arm group, with cages more frequently located at the anterior-center of the endplate. (C) Cage rotation in both groups, with an average rotation angle of 74 degrees in the C-arm-free group and 77 degrees in the C-arm group. The statistical analysis was done by the Mann-Whitney U test and a significant difference was set at p<0.05.

In the C-arm-free group, cages were predominantly positioned at the mid-center of the endplate, with 65% at the mid-center, followed by 32% at the anterior-center. Conversely, in the C-arm group, cages were more frequently placed at the anterior-center of the endplate, with 64% at the anterior-center and 22% at the mid-center (p=0.0063, Fisher’s exact test). The average cage rotation angle was 74 degrees in the C-arm-free group and 77 degrees in the C-arm group, with no significant intergroup difference (p=0.919, Figure 4).

Evaluation of complications

The C-arm-free group exhibited significantly fewer pedicle screw malpositions compared to the C-arm group (p=0.024), with one in the C-arm-free group and four in the C-arm group. The incidence of pedicle screw loosening, nonunion, dural tears, and surgical site infection rates did not significantly differ between the groups (Table 2). Notably, no cases of epidural hematoma, neurological deterioration, or cage back out were observed in either group.

**Table 2:** Surgical complications: C-arm-free group versus C-arm group The statistical analysis was done by Fisher's exact test and a significant difference was set at p<0.05.

Surgical complications	C-arm free	C-arm	p-value
Number of patients (N)	30	13	-
Pedicle screw malposition	1	4	0.024
Patients with pedicle screw loosening	2	4	0.350
Malunion (cases)	0	2	0.086
Dural tear (cases)	3	2	0.630
Post-operative infection (cases)	1	0	0.999

The ODI (%) at final follow-up

**Figure 5:**
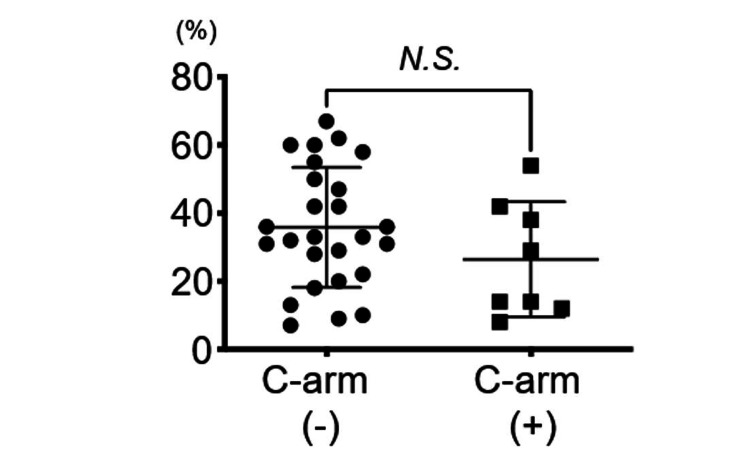
ODI (%) at final follow-up No significant differences were observed in the ODI (%) between the C-arm group and the C-arm-free group at the last follow-up. The statistical analysis was done by the Mann-Whitney U test and a significant difference was set at p<0.05. ODI: Oswestry Disability Index

At the final follow-up, the ODI percentages were 26.3 in the C-arm group and 35.8 in the C-arm-free group, revealing no significant differences (p=0.196) (Figure 5).

## Discussion

In the current study, we have compared radiologic and clinical outcomes of two techniques used in MIS-TLIF; C-arm-free and C-arm dependent techniques. Our findings demonstrated that although no clinical outcome difference was present, pedicle screw misplacement was significantly higher in C-arm-dependent MIS-TLIF. Additionally, there was no significant difference in operating time and blood loss among the two techniques. MIS-TLIF presents a compelling alternative to open TLIF. These methods appear to produce comparable clinical outcomes to open TLIF, showing similar improvements in ODI and Visual Analog Scale (VAS) scores during follow-up, as well as comparable fusion rates with no significant differences [8]. The advent of minimally invasive technology and instruments has heightened patients' preference for minimizing iatrogenic injury during and after surgery. Hence, contemporary technologies such as O-arm navigation, capable of delivering real-time intraoperative 3D images of the operative area to enhance the precision of pedicle screws and cage insertion, are increasingly being embraced nowadays [9]. The use of O-arm navigation in C-arm-free MIS-TLIF demonstrated comparable precision in pedicle screw placement and cage orientation to traditional C-arm techniques [10]. However, there is a paucity of studies comparing the outcomes of C-arm-free and C-arm-assisted MIS-TLIF procedures.

As far as we know, our research is the first to examine the differences in cage positioning between C-arm-free and C-arm techniques. Spinal fusion cages play a crucial role in maintaining intervertebral disc height, promoting bony fusion, and restoring lumbar lordosis in MIS-TLIF surgery [11]. Numerous studies have highlighted the significance of cage placement within the disc space as a critical factor influencing cage migration [12]. The incidence of cage migration varies from 0.8% to 23% in the literature [13]. Such migration of the cage can lead to direct compression of the neurological elements, the development of nonunion, and failure of the instrumentation [14].

The difference in cage location arose from the inability of the navigation system to monitor the cage rotation and such a difference had not any clinical importance. The importance of cage positioning within the disc space for achieving primary stability has been underscored by various biomechanical studies [15,16]. There is currently no universally agreed-upon standard for the optimal placement of cages during lumbar interbody fusion procedures. Some studies suggest that positioning the cage as anteriorly as feasible within the disc space not only reduces the risk of migration but also aids in achieving optimal lordosis [17]. However, biomechanical research highlights the potential benefits of placing the cage at the posterolateral endplate to minimize the likelihood of cage subsidence [18]. Interestingly, we found significant differences in cage placement between the two groups. In the C-arm-free group, cages were predominantly positioned at the mid-center of the endplate, while in the C-arm group, cages were more frequently placed at the anterior-center. This variation can be attributed to the use of banana cages, which are not compatible with navigation.

There were no significant differences in operative time and blood loss between both techniques, indicating that O-arm navigation can achieve similar efficiency as the C-arm method [19,20]. In contrast to our study, a study conducted by Peng et al. reported longer operative times with navigation-assisted procedures [6]. This discrepancy may be attributed to differences in surgical techniques, the learning curve of surgical staff, or variations in the navigation systems utilized [6]. Importantly, the use of O-arm navigation substantially reduces the surgeon's exposure to ionizing radiation, a critical occupational hazard in procedures utilizing fluoroscopy [2,21]. This benefit enhances the overall safety of the procedure for medical personnel [22]. In our study, the personnel in the operating room were not exposed to radiation at any phase of the procedure.

In our study, the C-arm group had a higher incidence of pedicle screw malpositioning (four cases) compared to only one case in the O-arm group (p=0.024). However, the difference in pedicle screw loosening between the groups was not statistically significant (p=0.208). The significant reduction in pedicle screw malpositioning in the O-arm group suggests that O-arm navigation may enhance surgical accuracy, which is directly linked to patient safety and the success of fusion procedures [23,24]. Numerous studies have corroborated this discovery, demonstrating that navigated techniques result in a notable decrease in the incidence of screw misplacement [25-27]. The findings from these studies revealed that 69-94% of the screws remained entirely within the pedicle during freehand technique, 28-85% during fluoroscopy, and 89-100% during CT navigation [27,28]. In navigation-assisted procedures, the ability to visualize axial scans provides enhanced anatomical detail compared to conventional C-arm imaging. Additionally, real-time insertion guidance allows for live monitoring of instrument placement, offering surgeons greater precision and confidence during the procedure [29].

Other surgical complications such as nonunion, dural tear, neurological deterioration, and surgical site infections did not show significant differences between the two groups. Both groups showed similar postoperative disability levels, as indicated by the ODI percentage at the final follow-up. This suggests that the choice between C-arm-free and traditional C-arm methods might not significantly impact patient recovery and long-term quality of life, making O-arm navigation a viable option for MIS-TLIF [30].

The study's limitations include its retrospective design, small sample size, and the need for longer follow-up to assess long-term outcomes. Navigation systems also have limitations such as calibration errors, sight obstruction, intraoperative changes, learning curves, cost and infrastructure, and potential for technology failure. Future research directions include conducting prospective, randomized trials to compare navigation-assisted versus C-arm-guided MIS-TLIF and evaluating the cost-effectiveness of navigation-assisted surgery.

## Conclusions

This study highlights that O-arm navigation significantly improves screw placement accuracy in MIS-TLIF, with acceptable cage placement, and clinically comparable outcomes to traditional methods. The reduction in radiation exposure with O-arm use emphasizes its promise for enhancing surgical team safety. These encouraging results advocate for the broader adoption of O-arm navigation in spinal fusion surgeries, marking a step forward in surgical precision and safety.

## References

[1] Ghauri MS, Reddy AJ, Tak N (2023). Utilizing deep learning for X-ray imaging: detecting and classifying degenerative spinal conditions. Cureus.

[2] Cloward RB (1953). The treatment of ruptured lumbar intervertebral discs by vertebral body fusion. I. Indications, operative technique, after care. J Neurosurg.

[3] Tanaka M, Zygogiannnis K, Sake N (2023). A C-arm-free minimally invasive technique for spinal surgery: cervical and thoracic spine. Medicina (Kaunas).

[4] Fan Y, Du JP, Liu JJ, Zhang JN, Qiao HH, Liu SC, Hao DJ (2018). Accuracy of pedicle screw placement comparing robot-assisted technology and the free-hand with fluoroscopy-guided method in spine surgery: an updated meta-analysis. Medicine (Baltimore).

[5] Zygogiannis K, Tanaka M, Sake N (2023). Our C-arm-free minimally invasive technique for spinal surgery: the thoracolumbar and lumbar spine-based on our experiences. Medicina (Kaunas).

[6] Peng P, Chen K, Chen H (2020). Comparison of O-arm navigation and microscope-assisted minimally invasive transforaminal lumbar interbody fusion and conventional transforaminal lumbar interbody fusion for the treatment of lumbar isthmic spondylolisthesis. J Orthop Translat.

[7] Gertzbein SD, Robbins SE (1990). Accuracy of pedicular screw placement in vivo. Spine (Phila Pa 1976).

[8] Vazan M, Gempt J, Meyer B, Buchmann N, Ryang YM (2017). Minimally invasive transforaminal lumbar interbody fusion versus open transforaminal lumbar interbody fusion: a technical description and review of the literature. Acta Neurochir (Wien).

[9] Kleck CJ, Johnson C, Akiyama M, Burger EL, Cain CJ, Patel VV (2018). One-step minimally invasive pedicle screw instrumentation using O-arm and stealth navigation. Clin Spine Surg.

[10] Lewis CS, Stone LE, Forseth KJ, Pham MH (2023). Minimally invasive C1-3 posterior spinal fusion with intraoperative O-arm navigation: 2-dimensional operative video. Oper Neurosurg (Hagerstown).

[11] Cheh G, Bridwell KH, Lenke LG, Buchowski JM, Daubs MD, Kim Y, Baldus C (2007). Adjacent segment disease followinglumbar/thoracolumbar fusion with pedicle screw instrumentation: a minimum 5-year follow-up. Spine (Phila Pa 1976).

[12] Hu YH, Niu CC, Hsieh MK, Tsai TT, Chen WJ, Lai PL (2019). Cage positioning as a risk factor for posterior cage migration following transforaminal lumbar interbody fusion - an analysis of 953 cases. BMC Musculoskelet Disord.

[13] Duncan JW, Bailey RA (2013). An analysis of fusion cage migration in unilateral and bilateral fixation with transforaminal lumbar interbody fusion. Eur Spine J.

[14] Tanaka M, Wei Z, Kanamaru A (2022). Revision for cage migration after transforaminal/posterior lumbar interbody fusion: how to perform revision surgery?. BMC Surg.

[15] Polly DW Jr, Klemme WR, Cunningham BW, Burnette JB, Haggerty CJ, Oda I (2000). The biomechanical significance of anterior column support in a simulated single-level spinal fusion. J Spinal Disord.

[16] Pimenta L, Turner AW, Dooley ZA, Parikh RD, Peterson MD (2012). Biomechanics of lateral interbody spacers: going wider for going stiffer. ScientificWorldJournal.

[17] Vialle EN, Ramos GZ, Hinojosa FL, Guiroy A, Rocha LG, Arruda AO (2022). Correlation between cage positioning and lumbar lordosis in transforaminal lumbar interbody fusion (TLIF). Rev Bras Ortop (Sao Paulo).

[18] Hueng DY, Chung TT, Chuang WH, Hsu CP, Chou KN, Lin SC (2014). Biomechanical effects of cage positions and facet fixation on initial stability of the anterior lumbar interbody fusion motion segment. Spine (Phila Pa 1976).

[19] Yeo I, Klemt C, Melnic CM, Pattavina MH, De Oliveira BM, Kwon YM (2023). Predicting surgical operative time in primary total knee arthroplasty utilizing machine learning models. Arch Orthop Trauma Surg.

[20] Gerdessen L, Meybohm P, Choorapoikayil S (2021). Comparison of common perioperative blood loss estimation techniques: a systematic review and meta-analysis. J Clin Monit Comput.

[21] Bakri A, Mehta K, Lance DR (2005). Sterilizing insects with ionizing radiation. Sterile Insect Technique: Principles and Practice in Area-Wide Integrated Pest Management.

[22] Ocloo J, Garfield S, Franklin BD, Dawson S (2021). Exploring the theory, barriers and enablers for patient and public involvement across health, social care and patient safety: a systematic review of reviews. Health Res Policy Syst.

[23] Bosma SE, Wong KC, Paul L, Gerbers JG, Jutte PC (2018). A cadaveric comparative study on the surgical accuracy of freehand, computer navigation, and patient-specific instruments in joint-preserving bone tumor resections. Sarcoma.

[24] Allam Y, Silbermann J, Riese F, Greiner-Perth R (2013). Computer tomography assessment of pedicle screw placement in thoracic spine: comparison between free hand and a generic 3D-based navigation techniques. Eur Spine J.

[25] Amiot LP, Lang K, Putzier M, Zippel H, Labelle H (2000). Comparative results between conventional and computer-assisted pedicle screw installation in the thoracic, lumbar, and sacral spine. Spine (Phila Pa 1976).

[26] Cui G, Wang Y, Kao TH (2012). Application of intraoperative computed tomography with or without navigation system in surgical correction of spinal deformity: a preliminary result of 59 consecutive human cases. Spine (Phila Pa 1976).

[27] Jing L, Wang Z, Sun Z, Zhang H, Wang J, Wang G (2019). Accuracy of pedicle screw placement in the thoracic and lumbosacral spines using O-arm-based navigation versus conventional freehand technique. Chin Neurosurg J.

[28] Gelalis ID, Paschos NK, Pakos EE (2012). Accuracy of pedicle screw placement: a systematic review of prospective in vivo studies comparing free hand, fluoroscopy guidance and navigation techniques. Eur Spine J.

[29] Rajasekaran S, Vidyadhara S, Ramesh P, Shetty AP (2007). Randomized clinical study to compare the accuracy of navigated and non-navigated thoracic pedicle screws in deformity correction surgeries. Spine (Phila Pa 1976).

[30] Zhang B, Ma JS, Feng P, Hu Y, Liu JL, Kong QQ (2023). Clinical efficacy of minimally invasive transforaminal lumbar interbody fusion (MIS-TLIF) in the treatment of II° lumbar isthmic spondylolisthesis: a retrospective cohort study. Medicine (Baltimore).

